# Changes in quality of life in treatment-resistant schizophrenia patients undergoing VR-assisted therapy for auditory verbal hallucinations: A content analysis

**DOI:** 10.1192/j.eurpsy.2023.330

**Published:** 2023-07-19

**Authors:** M. Beaudoin, S. Potvin, K. Phraxayavong, A. Dumais

**Affiliations:** 1 Psychiatry and addictology, University of Montreal; 2 Research Center, Institut universitaire en sante mentale de Montreal; 3 Faculty of Medicine, McGill University; 4 Research Center, Institut national de psychiatrie légale Philippe-Pinel, Montreal, Canada

## Abstract

**Introduction:**

VR-Assisted Therapy (VRT) for auditory verbal hallucinations has been demonstrated to have a significant impact on the symptoms, beliefs, and quality of life of patients with treatment-resistant schizophrenia. However, little is known about how these changes are implemented into their lives and on which aspects these improvements occur.

**Objectives:**

This study aimed to qualitatively explore changes in the quality of life of patients who underwent VRT in the context of an ongoing clinical trial.

**Methods:**

Ten consecutive patients enrolled in an ongoing clinical trial were assessed using semi-guided interviews before as well as 3 months after VRT. These encounters have been recorded and transcribed. Then, the content of the participants’ discourse was thoroughly analyzed, leading to the generation of an extensive theme grid. Each utterance was then coded by at least two members of the research team, and each disagreement was then discussed in a group format until a consensus was reached. As the cases were analyzed, the grid was adapted in a back and forth manner. New participants were included until data saturation occurred.

**Results:**

The content analysis allowed the identification of nine main themes representing different aspects of the patients’ quality of life: psychiatric symptomatology, identity, occupations, wishes, interpersonal relationships, lifestyle, psychiatric follow-up, life events, and attitudes/behaviors during the interview. Each theme was then subdivided into more specific codes. By analyzing the evolution of the frequency of each subtheme, it was observed that, following therapy, patients presented with less psychotic symptoms, which were also perceived more positively, a better self-esteem, more hobbies and projects, as well as an overall improved lifestyle and mood.

**Conclusions:**

Investigating how VRT impacts the patients’ quality of life allows for a deeper understanding of how people with treatment-resistant schizophrenia can achieve meaningful changes and move towards a certain recovery process.

**Disclosure of Interest:**

None Declared

